# Crop nutrient management using Nutrient Expert improves yield, increases farmers’ income and reduces greenhouse gas emissions

**DOI:** 10.1038/s41598-020-79883-x

**Published:** 2021-01-15

**Authors:** Tek B. Sapkota, Mangi L. Jat, Dharamvir S. Rana, Arun Khatri-Chhetri, Hanuman S. Jat, Deepak Bijarniya, Jhabar M. Sutaliya, Manish Kumar, Love K. Singh, Raj K. Jat, Kailash Kalvaniya, Gokul Prasad, Harminder S. Sidhu, Munmun Rai, T. Satyanarayana, Kaushik Majumdar

**Affiliations:** 1 International Maize and Wheat Improvement Center (CIMMYT), El Batan, Mexico; 2 International Maize and Wheat Improvement Center (CIMMYT), New Delhi, India; 3 International Rice Research Institute (IRRI), NASC Complex, New Delhi, 110012 India; 4 CGIAR Research Program on Climate Change, Agriculture and Food Security (CCAFS), CIAT-Bioversity Alliance, Cali, Colombia; 5 ICAR-Central Soil Salinity Research Institute (CSSRI), Karnal, Haryana India; 6 International Maize and Wheat Improvement Center (CIMMYT), CSSRI, Karnal, India; 7 CCS Haryana Agriculture University, Hisar, Haryana India; 8 International Maize and Wheat Improvement Center (CIMMYT), Borlaug Institute for South Asia (BISA), Ludhiana, Punjab India; 9 International Maize and Wheat Improvement Center (CIMMYT), Borlaug Institute for South Asia (BISA), Pusa, Samastipur, Bihar India; 10 International Plant Nutrition Institute (IPNI), Gurgaon, Haryana 122001 India; 11 African Plant Nutrition Institute (APNI), & Univerśite Mohammed VI Polytechnique, Benguérir, Morocco

**Keywords:** Climate-change mitigation, Environmental impact

## Abstract

Reduction of excess nutrient application and balanced fertilizer use are the key mitigation options in agriculture. We evaluated Nutrient Expert (NE) tool-based site-specific nutrient management (SSNM) in rice and wheat crops by establishing 1594 side-by-side comparison trials with farmers’ fertilization practices (FFP) across the Indo-Gangetic Plains (IGP) of India. We found that NE-based fertilizer management can lower global warming potential (GWP) by about 2.5% in rice, and between 12 and 20% in wheat over FFP. More than 80% of the participating farmers increased their crop yield and farm income by applying the NE-based fertilizer recommendation. We also observed that increased crop yield and reduced fertilizer consumption and associated greenhouse gas (GHG) emissions by using NE was significantly influenced by the crop type, agro-ecology, soil properties and farmers’ current level of fertilization. Adoption of NE-based fertilizer recommendation practice in all rice and wheat acreage in India would translate into 13.92 million tonnes (Mt) more rice and wheat production with 1.44 Mt less N fertilizer use, and a reduction in GHG of 5.34 Mt CO_2_e per year over farmers’ current practice. Our study establishes the utility of NE to help implement SSNM in smallholder production systems for increasing crop yields and farmers’ income while reducing GHG emissions.

## Introduction

In recent years, the potential to mitigate climate change by improving nutrient use efficiency (NUE) in croplands has received considerable attention in the agricultural research and policy agendas^1–3^. The use of chemical fertilizers, nitrogen (N) in particular, in crop production is at the center of managing both food security and environmental problems^4–6^. Enhancing crop yields through increased use of nutrients is essential to meet current as well as future food demand^7^. On the other hand, because fertilizer application in croplands is a major source of anthropogenic nitrous oxide (N_2_O) emissions^8^, reducing greenhouse gas (GHG) emissions through proper fertilizer management is essential to address agriculture’s contribution to climate change^2^. Moreover, excess and improper use of nutrients in crop production have large cost implications for the farmers^9^. Therefore, improving NUE in croplands provides the opportunity to address the triple challenge of food security, farmers’ livelihood and environmental protection, globally.

Site-Specific Nutrient Management (SSNM) involves optimizing nutrient inputs considering demand (plant needs) and supply (from soils indigenous sources) of the nutrients according to their variation in time and space thereby ensuring field-specific nutrient management in a particular cropping season^10,11^. Various technologies and practices such as Chlorophyll Meter^12^, Leaf Color Chart^13^, GreenSeeker^14^ and decision support systems for instance Nutrient Expert (NE) (http://software.ipni.net) and Rice Crop Manager (http://cropmanager.irri.org/home) are available for helping farmers to implement SSNM and improve NUE^9,15^. The NE tool was developed to implement crop nutrient management specific to farmers' fields with or without a soil test^16^. Although a few studies have evaluated the agronomic and environmental performance of NE^17–19^, it has not been evaluated on a large number of farms with varying agro-climatic conditions and across various levels of crop intensification. Farmers’ participatory trials are useful to assess the utility of the tool and also enable farmers to make informed decisions for crop nutrient management.

This study presents results from a large number of on-farm participatory trials (1594-paired data) comparing farmers’ fertilizer practices (FFP) vs NE-based nutrient management in terms of fertilizer inputs, yields, economic returns, and GHG emissions in rice and wheat fields in India. Rice and wheat are the major crops grown in India and consume 50% of the fertilizer used in the country^20^. The study in India was particularly important because India consumes 14% of total fertilizer use globally^20^ but its NUE is one of the lowest in the world^21^. This is mainly driven by imbalanced and inadequate use of nutrients given the skewed government's subsidy on nitrogenous fertilizer than on other nutrients^22^. The results of this study provide rich information to the agriculture and fertilizer policymakers to enable them to design fertilizer use and distribution policies and farmers' support programs in the IGP and other parts of the country.

## Results

### Fertilizer input and crop yield

**Figure 1:**
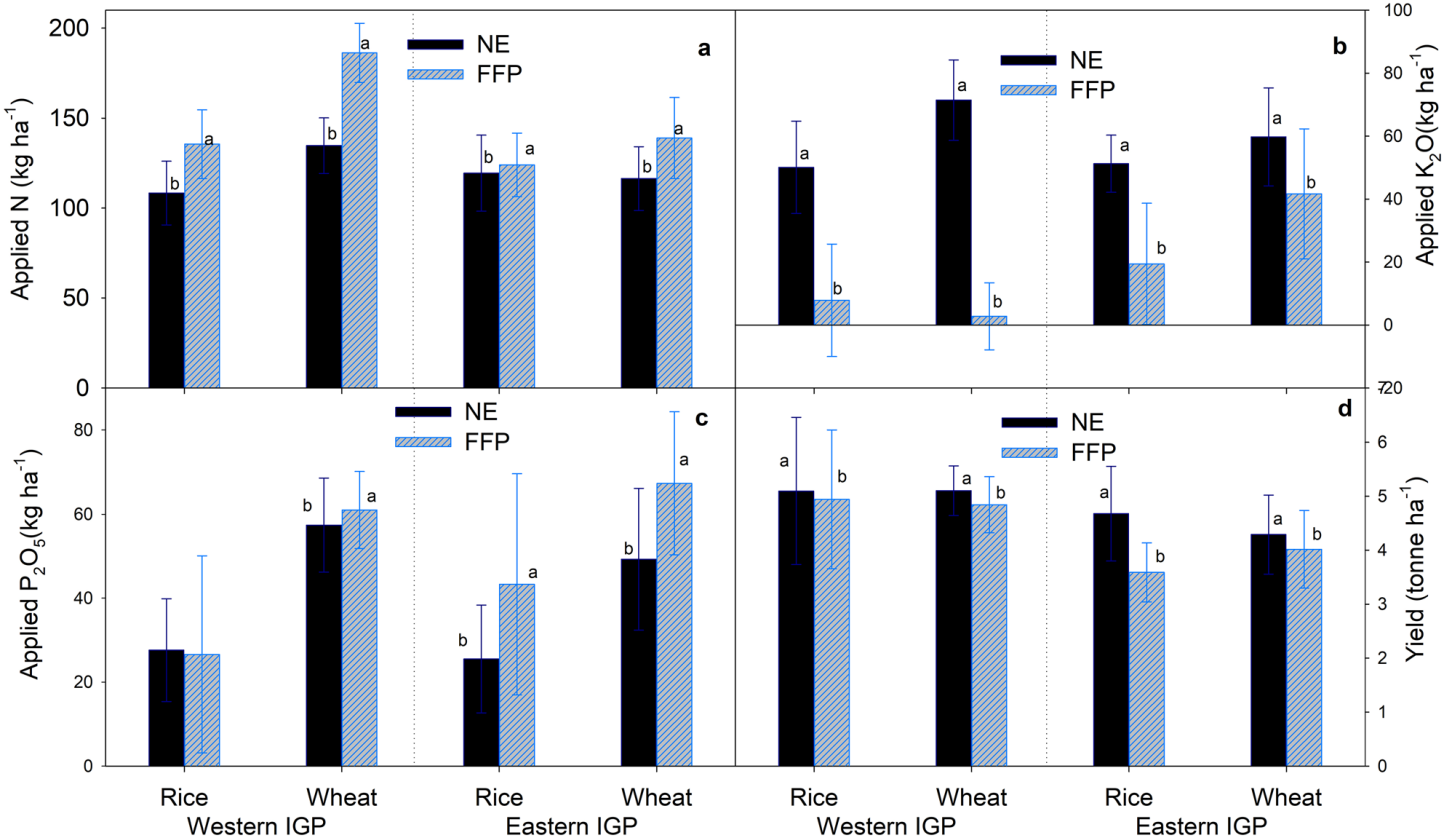
Rate of nitrogen (**a**), potash (**b**) and phosphorus (**c**) application and grain yield (**d**) from rice and wheat under Nutrient Expert (NE) and Farmers’ Fertilizer Practices (FFP) in the study areas. Values are average of all the comparison trials over the study period. Within each pair, bars bearing different lowercase letters are significantly different from each other based on the paired t-test (p = 0.05). The error bar shows the standard deviation. IGP = Indo-Gangetic Plains.

The use of the NE tool significantly reduced the amount of nigrogen (N) use in both rice and wheat crops compared to the FFP (Fig. 1a). Although potash (K_2_O) input was significantly higher under NE than under FFP (Fig. 1b), phosphorus (P_2_O_5_) input was also significantly lower under NE-based recommendations than FFP, except for rice in Western IGP (Fig. 1c). The N, P_2_O, and K_2_O application rates in each comparison trial in rice and wheat fields under NE and FFP in the study areas are presented in Supplementary Figure S1. Farmers in the Western IGP were either applying high P_2_O_5_ rates in rice cultivation or none at all. We also observed that many farmers in the IGP region were not using K_2_O on rice and wheat crops.

**Figure 2:**
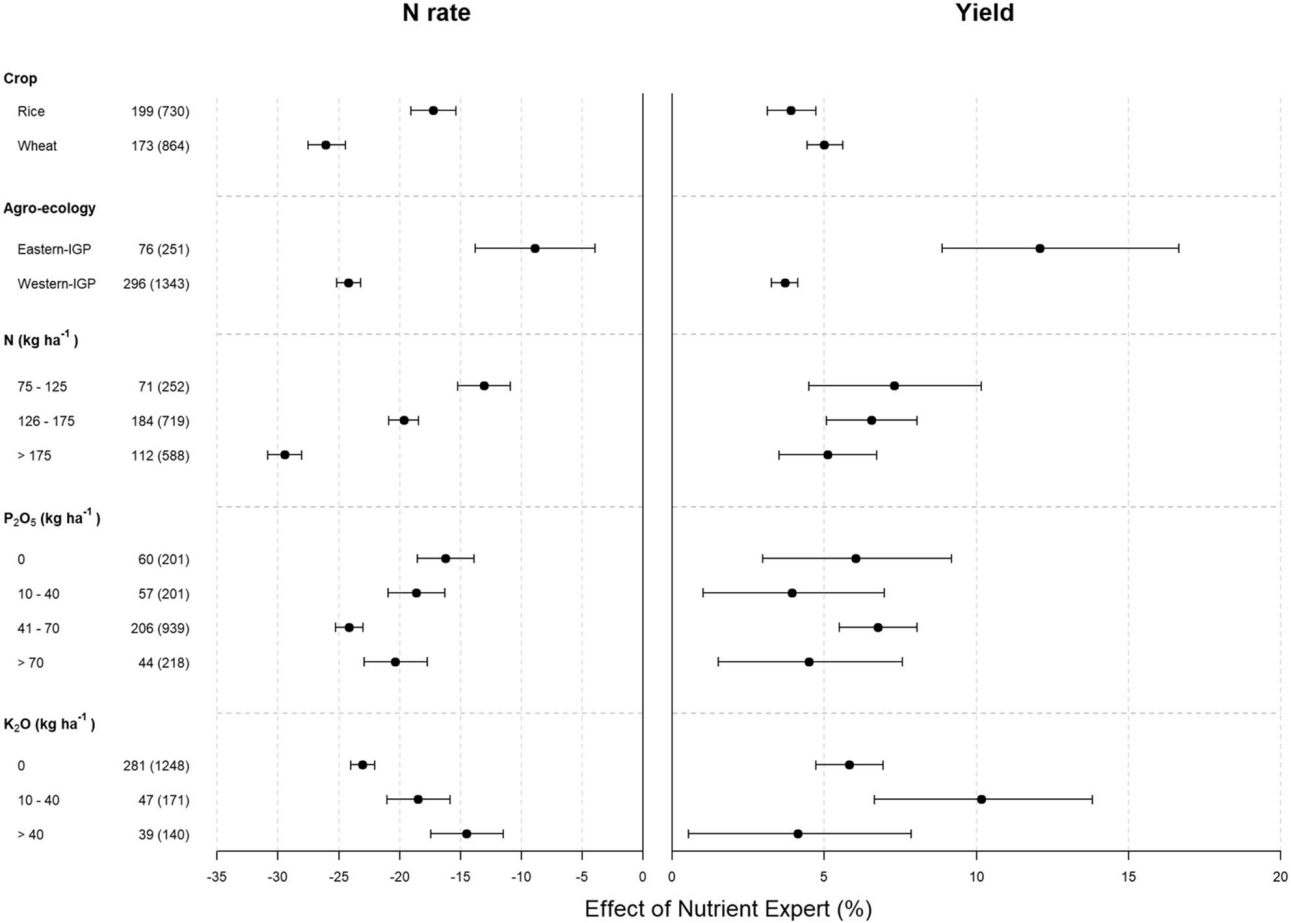
Percent change in nitrogen use rate (left panel) and grain yields (right panel) due to NE-based fertilizer management compared to FFP segregated by crop types, agro-ecological zones and farmers’ fertilizer application rates. In the Y-axis, the numbers outside parentheses denote the number of villages and the ones inside parentheses show the number of pairs analyzed. Error bars indicate 95% confidence intervals (CI). The changes are considered significant when 95% CI does not overlap zero, and the effects of categories within a parameter are significantly different when their 95% CI do not overlap with each other. IGP = Indo-Gangetic Plains.

The NE-based recommendation significantly reduced N application over the FFP in the wheat crop more than in rice, and in the Western IGP more than in the Eastern IGP (Fig. 2, left panel). The effect of NE in reducing N application over the FFP was influenced by farmers’ fertilizer application rates. The reduction in N rate by NE over FFP was higher in the cases where farmers’ N application rate was higher and the effect gradually reduced as the rate of farmers’ N application decreased (Fig. 2, left panel). Similarly, the effect of NE in reducing the N application rate was higher where farmers were applying between 41 and 70 kg P_2_O_5_ ha^−1^ than in the cases where farmers were not applying it or were applying smaller quantities, i.e., < 40 kg of P_2_O_5_ ha^−1^. N reduction was also higher in the cases where farmers were not applying K_2_O than in the cases where farmers were applying K_2_O (Fig. 2, left panel).

Overall, NE-based fertilizer management significantly increased the yield of both rice and wheat in both agro-ecologies as compared to FFP (Fig. 1d). However, the increment was highest in rice in Eastern IGP. When separated by agro-ecology, the yield increment due to NE was higher in Eastern IGP than in Western IGP (Fig. 2, right panel). Although the yield response to NE-based fertilizer management was not significant under different rates of fertilizer (N, P_2_O_5_ and K_2_O) application by farmers, yield improvement due to NE was comparatively high where farmers used less K_2_O (Fig. 2, right panel).

Out of 135 pair comparisons in the rice fields of Eastern IGP, NE yielded higher than FFP in all cases, with less N input than FFP in 49% of cases and with more N input than FFP in 50% of cases (Supplementary Fig. S2, upper left). In the case of wheat in this agro-ecology and compared to FFP, NE increased crop yield in 78% of cases out of 116 pair comparisons, in 65% of cases with reduced N input and in the remaining 13% of cases with increased N input (Supplementary Fig. S2, lower left). Here, compared to FFP, NE reduced yield in 20% of cases mostly with reduced N input. In Western IGP, on the other hand, compared to FFP, NE increased rice and wheat yield in 83% out of 595 pair comparisons and in 91% out of 748 pair comparisons, respectively, mostly with reduced N input (Supplementary Fig. S2, right panels).

### Greenhouse gas emissions

**Figure 3:**
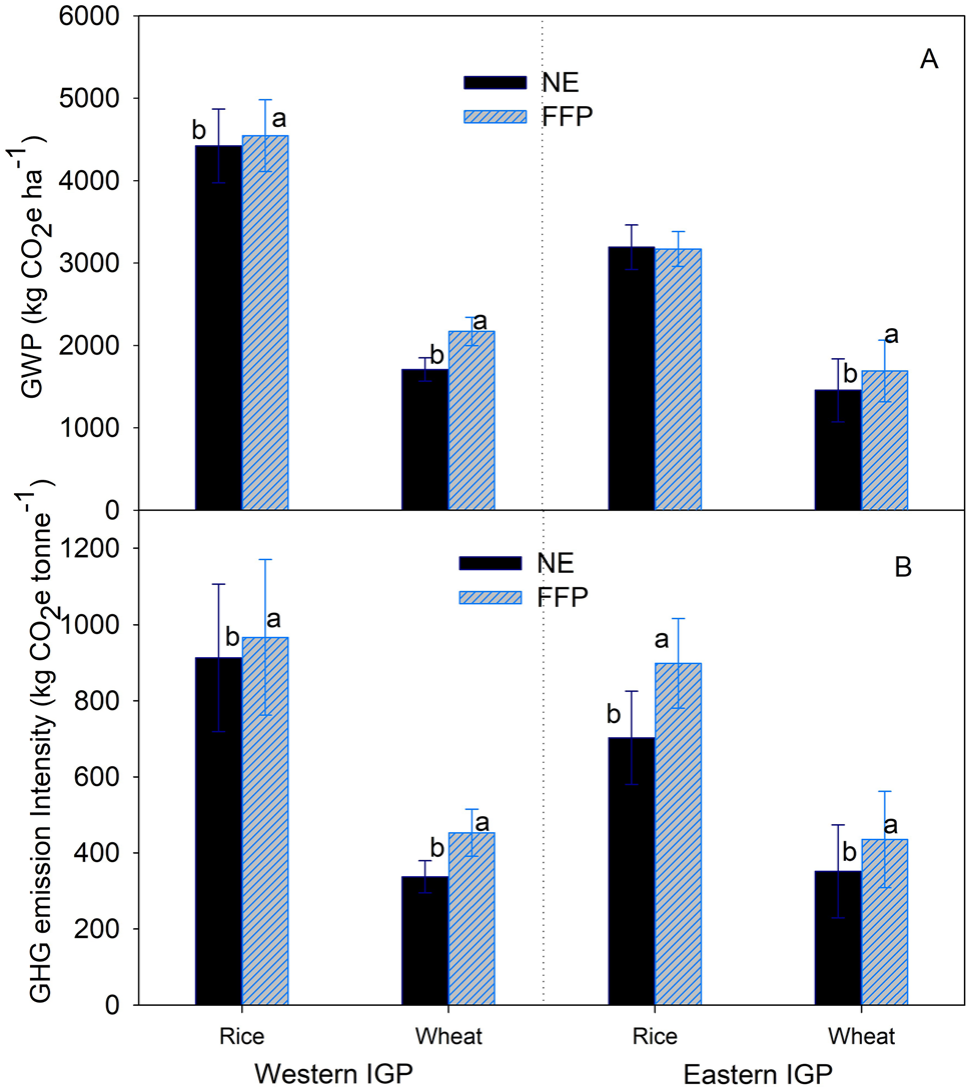
Total global warming potential (**A**) and emission intensity (**B**) from rice and wheat production under NE and FFP in the study areas. Values are average of all the comparison trials over the study period. Within each pair, bars bearing different lower case letters are significantly different from each other based on a paired t-test (p = 0.05). Error bar shows the standard deviation. IGP = Indo-Gangetic Plains.

The farm gate GWP from rice production in the study area ranged from 2463 to 5482 kgCO_2_e ha^−1^ whereas that from wheat production ranged from 287 to 2463 kgCO_2_e ha^−1^. Similarly, GHG emission intensity of rice ranged from 509 to 1606 kgCO_2_e tonne^−1^ grain yield and that of wheat ranged from 71 to 769 kgCO_2_e tonne^−1^ grain yield. Total GWP and GHG emission intensity of rice and wheat production was significantly lower under NE-based fertilizer management than under FFP in both agro-ecologies (Fig. 3A,B).

**Figure 4:**
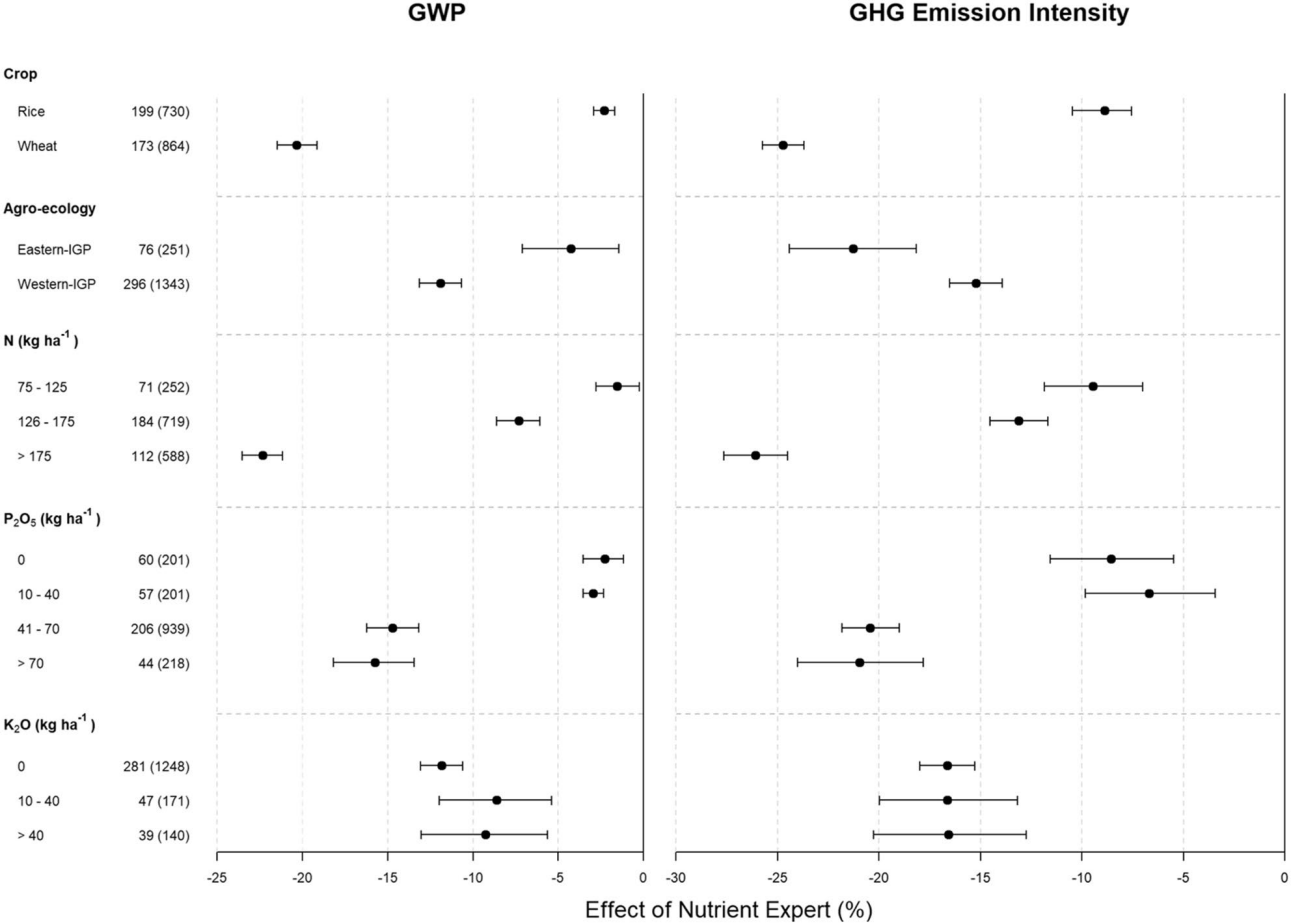
Percent change in global warming potential (GWP, left panel) and emission intensity (right panel) due to NE-based fertilizer management compared to FFP segregated by crop types, agro-ecological zones and farmers’ fertilizer application rates. In the Y-axis, the numbers outside parentheses denote the number of villages and the ones inside parentheses show the number of pairs analyzed. Error bars indicate 95% confidence intervals (CI). The changes are considered significant when 95% CI does not overlap zero, and the effects of categories within a parameter are significantly different when their 95% CI do not overlap with each other. IGP = Indo-Gangetic Plains.

The NE-based fertilizer management reduced more GWP in wheat than in rice and more in Western IGP than in Eastern IGP (Fig. 4, left panel). When separated by farmers’ fertilizer rate, the effect of NE-based fertilizer recommendations in reducing total GWP was significantly smaller with lower applications of N and P_2_O_5_ (< 40 kg P_2_O_5_ ha^−1^) than in the cases where farmers were applying higher rates of these fertilizers (Fig. 4, left panel). Similarly, the effect of NE in reducing GHG intensity was higher in wheat than in rice and higher in Eastern IGP than in Western IGP (Fig. 4, right panel). The effect of NE in reducing total GHG emission intensity was significantly smaller with lower applications of N and P_2_O_5,_ (< 40 kg P_2_O_5_ ha^−1^) than in cases where farmers were applying higher rates, i.e., > 175 kg N and > 40 kg P_2_O_5_ ha^−1^ (Fig. 4, right panel).

### Cost–benefit of change in fertilizer rate

**Figure 5:**
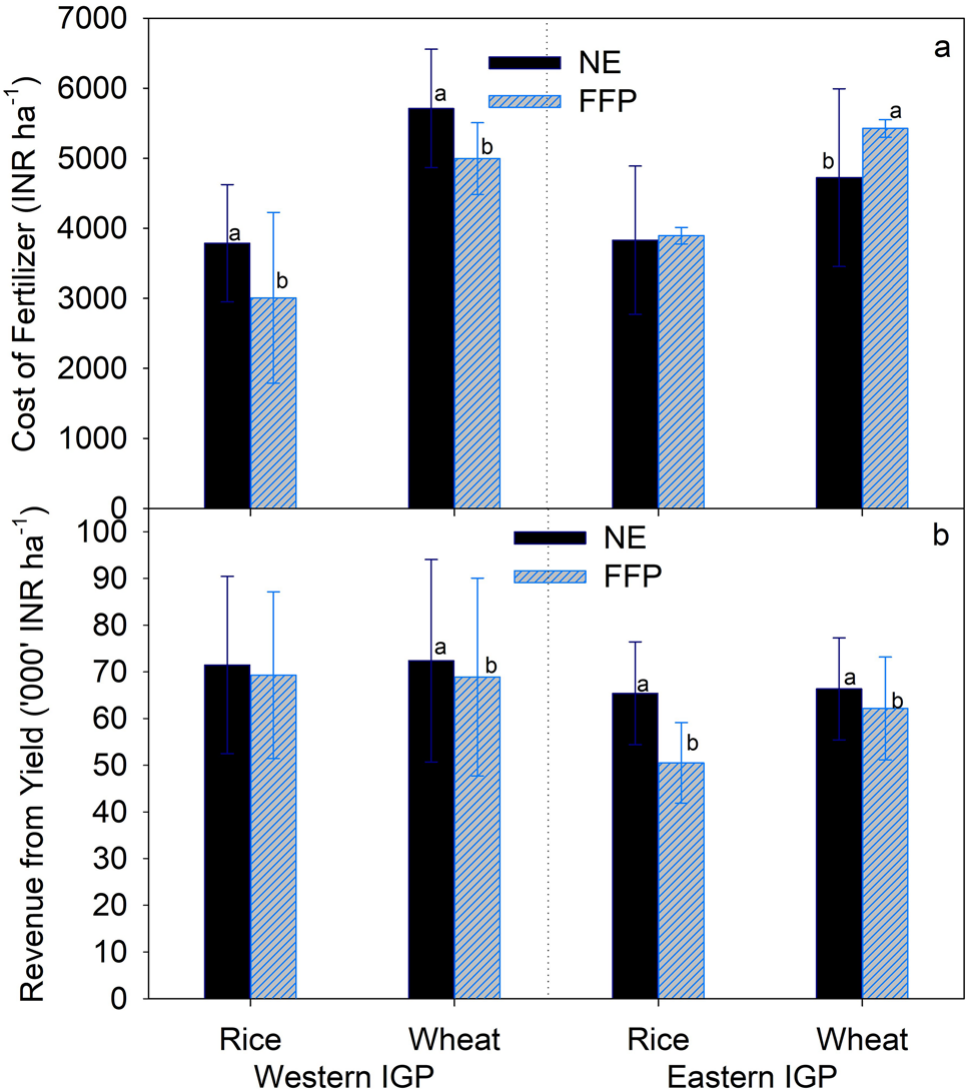
Cost of fertilizer and income from yield in rice and wheat production under Nutrient Expert (NE) and Farmers’ Fertilizer Practice (FFP) in the study areas. Values are average of all the comparison trials over the study period. Within each pair, bars bearing different lowercase letters are significantly different from each other based on paired t-test (p = 0.05). Error bar shows standard deviation. IGP = Indo-Gangetic Plains.

**Figure 6:**
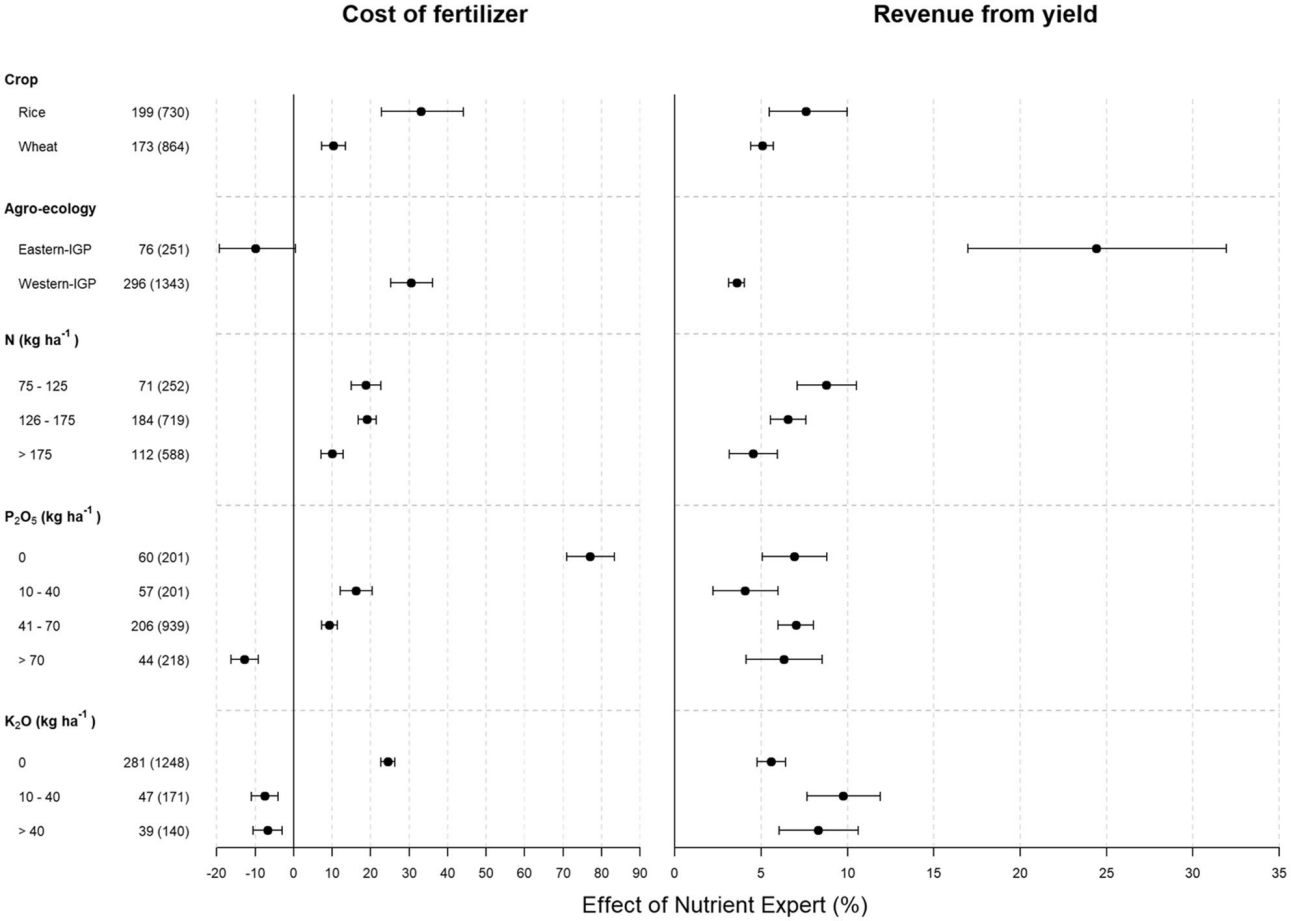
Percent in fertilizer cost (left panel) and revenue from yield (right panel) due to NE-based fertilizer management compared to FFP segregated by crop types, agro-ecological zones and fertilizer application rates. In the Y-axis, the numbers outside parentheses denote the number of villages and the ones inside parentheses show the number of pairs analyzed. Error bars indicate 95% confidence intervals (CI). The changes are considered significant when 95% CI does not overlap zero, and the effects of categories within a parameter are significantly different when their 95% CI do not overlap with each other. IGP = Indo-Gangetic Plains.

The cost of fertilizer application under NE-based recommendations in rice and wheat crops was largely affected by the FFP in the different agro-ecological zones. NE significantly increased the cost of fertilizer for both rice and wheat in Western IGP (Fig. 5a). In Eastern IGP, the cost of fertilizer for rice was not significantly different between NE and FFP, whereas that for wheat was significantly higher under FFP (Fig. 5a). The percent increment in fertilizer cost due to NE over FFP was higher in rice than in wheat (Fig. 6, left panel). When separated by agro-ecologies, compared to FFP, NE significantly increased cost of fertilizer in Western IGP but reduced it in Eastern IGP, although not significantly. Compared to FFP, NE slightly increased the total cost of fertilizer in cases where farmers’ N rate was < 175 compared to cases where farmers’ N rate was > 175 kg ha^−1^ (Fig. 6, left panel). The total cost of fertilizer due to NE significantly increased in cases where farmers did not apply any P_2_O_5_ or K_2_O, and the total cost actually decreased in cases where farmers applied > 70 kg P_2_O_5_ and > 10 kg K_2_O ha^−1^ (Fig. 6, left panel).

Except for rice in Western IGP, NE significantly increased the gross income from crop production compared to FFP (Fig. 5b). When separated by agro-ecological zones, the revenue increase due to NE over FFP was significantly higher in Eastern IGP than in Western IGP (Fig. 6, right panel). Similarly, the percentage of revenue increase from NE over FFP was higher in cases where farmers’ N application rate was lower (N < 125 kg N ha^−1^) than in cases where farmers’ N application rate was higher (N > 175 kg N ha^−1^).

## Discussion

### Use of the nutrient expert tool for SSNM

This study presented the benefits of using the NE tool for SSNM in rice and wheat crops in two different agro-ecological zones in India. The NE-based nutrient management not only increased the grain yield of rice and wheat crops but also decreased the total N and P_2_O_5_ applied in the fields (Fig. 1). The results of increased yields and decreased N rates through NE-based nutrient management are in agreement with other findings in the region^19^ that also reported increased yield and reduced consumption of N and P_2_O_5_ through NE-based fertilizer recommendation. Increased crop yield and reduced fertilizer consumption by NE can be attributed to increased NUE as NE gives dynamic fertilizer recommendation based on growing conditions, soil indigenous nutrient supply and residual nutrients from previous crops thereby minimizing the loss of nutrients. Through on-farm comparison of various nutrient management strategies, Sapkota et al.^19^ reported significant improvement in the use efficiency of N and P_2_O_5_ through NE based fertilizer management in wheat thereby increasing yield and profitability. In addition, a more balanced nutrition in NE might have led to increases in NUE through more vigorous plant growth and greater tolerance against biotic and abiotic stresses. In general, farmers do not apply K fertilizer resulting into imbalanced nutrients, which ultimately reduces the efficiency of other applied nutrients. Xu et al.^23^ also reported increased NUE and crop yield due to NE over farmers’ practice of fertilizer management in spring maize in Northeast China.

The magnitude of the effect of NE in improving NUE thereby decreasing the use of fertilizers and/or increasing yields varied by crops, agro-ecology and farmers’ current fertilizer rates. The regional variations in N reduction and yield gains (Fig. 2) were largely influenced by the current level of crop intensification and yield. The net gain through NE-based fertilizer recommendations is probably due to the increased partial factor productivity of N, which can be attributed to the balanced use of nutrients in rice and wheat crops. The majority of rice and wheat farmers in the IGP and elsewhere in South Asia apply N fertilizer following blanket recommendations based upon crop response data averaged over large geographic areas^24^. Crop fertilization following such blanket recommendations results in under-fertilization in some areas and over-fertilization in others. The NE-based recommendations help to overcome this problem by considering field conditions, management practices and crop characteristics for nutrient application.

This study presented interesting results on N rate change and yield response across the agro-ecologies. Most farmers (64% of rice growers and 77% of wheat growers) realized yield increases despite the reduction in N application on both rice and wheat crops (Fig. S2). Only about 15% of rice growers and 12% of wheat growers experienced yield losses due to NE but with decreased N application, probably because these farmers were applying N beyond the economic optimum. Increased N application had positive yield impacts for 27% of rice and 7% of wheat growers mainly in Eastern IGP (Fig. S2). This shows an opportunity to close yield gaps in low-input areas by NE. Further, the percentage of N reduction by NE under high N rate in farmers’ fields (Fig. 2, left panel) with equal yield improvement (Fig. 2, right panel) suggests that the benefit of NE over FFP comes by both reducing N rate and improving yield in high-input areas, and mainly by increasing yield in low-input areas.

Overall, NE-based fertilizer management in rice–wheat systems can reduce N fertilizer by ca. 18% (ca. 10% in Eastern IGP and ca. 25% in Western IGP; Fig. 2, left panel) without compromising yield (Fig. 2, right panel). Our analysis further shows that SSNM through NE increased rice and wheat production by about 4–12% in India (Fig. 2, right panel). These results reveal that NE-based SSNM has great potential for improving yields and NUE in rice and wheat crops to close the existing yield gaps and reduce excess N application. Moreover, efficient N fertilizer management strategies through adoption of NE-based recommendations could be one of the sustainable intensification pathways for the rice–wheat system in IGP and similar agro-ecologies in the region and beyond.

### Cost implications of the NE use

This study showed that NE-based fertilizer recommendation has both positive and negative impacts on the total cost of fertilizer use. More than 50% of farmers (55% in rice and 51% in wheat) have experienced increase in total cost of fertilizer over FFP (Supplementary Fig. S3). This also varied across the agro-ecological zones, again mainly due to different levels of crop intensification. Many farmers in the study areas either avoid or practice low application of P_2_O_5_ and K_2_O. NE balances the fertilizer use with adequate application of P_2_O_5_ and K_2_O, which increased the total cost of fertilizer use despite the reduction in N application (Fig. 6, left panel). However, we also observed that, compared to FFP, NE reduced the total fertilizer cost for many farmers (45% in rice and 49% in wheat). These farmers were already using some amount of P_2_O_5_ and K_2_O, and therefore, the cost decrease came mainly from reduced N application under NE.

Most farmers experienced yield gain and, therefore, higher income from grain yield despite the increase or decrease in total fertilizer cost (Fig. 6, right panel, and Fig. S2). In cases where yield gain was achieved due to increased fertilizer use, the increase in fertilizer cost was compensated by the yield gains. Few farmers in our study have received double gain i.e. decrease in fertilizer cost as well as yield gains. This is probably due to balanced fertilization with adequate application of potassium under NE-based fertilizer management that improved the NUE and increased the grain yields, thereby resulting in positive net returns. Larger revenue gain than the fertilizer cost indicates a large yield gap and huge potential to close this gap through better fertilizer management. Our results are in agreement with Xu et al.^23^ who reported that increase in gross return above fertilizer cost due to NE in spring maize was mainly due to increase in grain yield.

### GHG mitigation potential

This study showed there is a large potential for reducing excess N from rice and wheat fields with the use of NE-based fertilizer recommendations (Fig. 1a). Application of N fertilizer is typically a main driver of N_2_O fluxes from rice–wheat systems^25^. The GHG emission reduction due to NE-based fertilizer management was higher in wheat than in rice (Fig. 4), mainly because of two reasons. Firstly, farmers generally apply higher doses of N fertilizer in wheat than in rice (Fig. 1a) and N reduction due to NE-based fertilizer management was higher in wheat than in rice (Fig. 2, left panel). This results in more GHG reduction in wheat. Secondly, the fertilizer-induced field emissions of N_2_O would be higher in upland crops such as wheat than in lowland crops such as rice^26^. Therefore, even with same level of fertilizer N reduction through NE, the percentage of GHG emission reduction would be higher in wheat than in rice. This emission reduction potential also varied spatially depending on the current level of fertilizer use (Fig. 4, left panel). Reduction in both GWP as well as GHG emission intensity by NE was higher in cases where farmers were applying higher rates of N and P_2_O_5_ (Fig. 4). This was mainly due to the reduction in fertilizer use by NE in these cases (Fig. 2, left panel) and subsequently, the reduction in fertilizer-induced emissions of N_2_O and CO_2_.

The emission intensity in both crops decreased under NE-based recommendations (Fig. 3, upper panel) due to the partial or combined effect of the reduction in N application and yield gain by NE (Figs. 1 and 2). NE reduced GHG emission intensity more in wheat than in rice (Fig. 4, right panel). This is because NE resulted into higher reduction in N rates and higher yield increments in wheat than in rice (Fig. 2). Similarly, NE reduced GHG emission intensity more in Eastern IGP than in Western IGP (Fig. 4, right panel) mainly because NE resulted in larger yield gains in Eastern IGP compared to Western IGP (Fig. 2, right panel). These results demonstrate the importance of NE for closing yield gaps in low-input production systems. Magnitude of GHG reduction by NE in our study (ca. 2.5% in rice, and 12–20% in wheat) was lower than reported by Zhang et al.^27^ (ca. 45%) from winter wheat in North-central China. This was mainly because farmers in North-Central China commonly apply higher dose of N (> 300 kg N ha^−1^)^27,28^. Thus, the magnitude of N reduction through adoption of NE is higher in such areas with over-fertilization and therefore higher magnitude of fertilizer-induced GHG savings.

### Implications of NE-based nutrient management

Mineral fertilizers play an important role in increasing crop production and securing food security of growing population. However, excessive or imbalanced use of fertilizer not only increase the production cost to farmers but also contributes to the environmental pollution. Therefore, in intensive crop growing area such as Indian IGP, N fertilizer must be applied judiciously to balance optimum yield against the cost of fertilizer and the negative environmental effects of excess N application. Agriculture is the second largest source of GHG emissions in India, accounting for ~ 18% of gross national emissions. Identifying high-yield low-emission pathways for the country’s cereal production is key for reducing agriculture’s contribution to total GHG emissions^24^. India recently declared a voluntary goal of reducing the emission intensity of its gross domestic product by 35% over the 2005 level, by 2030 (India’s NDC submitted to UNFCCC). Reduction in excess nutrient application and balanced fertilizer use are the key mitigation options in Indian agriculture^29,30^. This option can contribute to reducing large amounts of GHG emissions from the agriculture sector including gains in crop yield and income for most farmers. Soil test-based fertilizer recommendations are difficult to use for smallholder farms in South Asia because of constraints such as available testing facilities, farmers’ access to such facilities, cost, and timeliness. Given the situation, a science-based, reliable and practically feasible site-specific fertilizer recommendation method is required to respond to the low NUE caused by imbalanced fertilization practices. Decision support systems (DSS) are nowadays progressively being used to facilitate application of improved nutrient management practices in the farmers’ fields.

Thus, scaling the use of NE-based SSNM can partially address the challenge of increasing food production to meet the growing food demand and reducing agricultural emissions particularly in the area where crop yield gaps and agricultural emissions are high. NE-based nutrient recommendation can be scaled-up through government extension systems and schemes (e.g. Soil Health Card Scheme of India: https://www.soilhealth.dac.gov.in/). Based on authors’ experience in the region, NE is easy to learn by farmers, can be used through their android cell phones. Many progressive farmers in the study area are already using NE not only on their farm but also in the farm of fellow farmers.

The implications of NE-based fertilizer management in terms of yield, N consumption and GHG emissions are tremendously high in countries like India. In 2016–2017, India produced 109.7 and 98.51 Mt of rice and wheat, respectively (https://eands.dacnet.nic.in/). If our observed on-farm yield increases of rice and wheat through NE-based fertilizer management over FFP represent the total rice and wheat area in India, this will translate into the production of 8.5 and 5.4 Mt additional rice and wheat, respectively, without additional production costs. Annual N fertilizer consumption in India was about 17.4 Mt in 2016–2017^31^. Assuming 50% of this total N is used for rice and wheat production^20^, estimated N fertilizer savings due to NE-based fertilizer management in rice and wheat in India will be about 1.44 Mt with huge implications on costs and GHG savings. Through a bottom-up analysis using a large number of datasets in India, Sapkota et al. calculated fertilizer-related emissions from rice and wheat to be 558 and 775 kg CO_2_e per ha, respectively^29^. If our results of GHG emission savings of 2.5% in rice and 20% in wheat due to NE-based fertilizer management could be achieved in all rice and wheat areas in the country, this would translate into GHG savings of 5.2 Mt CO_2_e, i.e., 0.61 Mt CO_2_e from rice and 4.63 Mt CO_2_e from wheat. However, this would also increase the consumption of K_2_O with huge implications for the production and import of K fertilizer and associated costs and this trade-off warrants further study. We conducted this research both in high-input (Western IGP) and low-input (Eastern IGP) production systems in the major rice–wheat belt of India and covered a sufficiently large number of farmers (ca. 1600 pair comparisons) to make it representative of major rice–wheat growing areas in the region. Given the level of implications in terms of yields, total N, P_2_O_5_ and K_2_O consumptions and GHG emissions, NE-based fertilizer management certainly merits further scientific investigation and policy consideration.

## Conclusion

This study evaluated NE-based site-specific nutrient management vis-à-vis farmers’ fertilizer practice in rice and wheat in both high-input and low-input production systems across the rice–wheat belt of India through large numbers of on-farm comparison trials. Overall, NE-based recommendations reduced N input by 15–35%, increased grain yield by 4–8% and reduced global warming potential by 2–20%. The study also shows that NE-based SSNM is more important for closing the yield gap in low-intensive systems and decreasing nutrient input and minimizing nutrient loss high-intensive systems. Adoption of NE-based site-specific nutrient management across all rice and wheat growing areas in India would translate into additional grain production of 13.92 Mt, N consumption reduction of 1.44 Mt and total GHG savings of 5.24 Mt CO_2_e per year with some additional use of K fertilizer. In smallholder production systems, where soil testing of each field is nearly impossible, a simple decision support tool such as NE could be helpful to promote site-specific nutrient management contributing to both food security and environmental sustainability goals.

## Methods

### On-farm comparison trials

We conducted this study in farmers’ fields in the states of Punjab, Haryana and Bihar in India (Fig. S4). Punjab and Haryana represent high-input production systems typical of the Western Indo-Gangetic Plains (IGP), whereas Bihar represents relatively low-input production systems typical of the Eastern IGP. In general, intensive mechanized production makes the cereal systems of IGP GHG emission intensive. The Western IGP are characterized by semi-arid climate with mean annual rainfall varying from 544 to 970 mm. The climate in Eastern IGP is characterized by hot and humid summers and cold winters, with an average annual rainfall of 1350 mm, 70% of which falls between July to September. An overview of the agro-ecological conditions of the study sites are given in Supplementary Table 1.

We conducted on-farm comparison trials for four years during the 2013–2014 to 2016–2017 cropping seasons. Altogether, we conducted 1594 pair comparison trials (1094 in Haryana, 245 in Punjab and 251 in Bihar), 730 trials for rice and 864 trials for wheat. Each trial had two paired plots–one with a fertilizer recommendation determined by NE and one with FFP. We used basic information about the plots, such as soil characteristics, yield and nutrients applied to previous crops, together with information about the present crop and the target yield to estimate nutrient recommendation from NE software (http://software.ipni.net). NE estimates the attainable yield utilizing the information provided by farmers about growing conditions, determines the nutrient balance in the cropping system based on yield and nutrients applied to previous crops, and combines such information with soil characteristics to predict the crop response to N, P and K, and generates nutrient recommendations specific to that field^16^. The plot size ranged from 1000 to 2000 m^2^ in Haryana and Punjab, and from 500 to 1500 m^2^ in Bihar. All practices, except fertilizer management, were similar for paired plots within the comparison trials. The participating farmers primarily managed the plots. The researchers consulted with farmers to calculate NE-based recommendations using the Nutrient Expert tool and collected relevant data from the trials.

### Crop management in the field

Most farmers adopted intensive tillage practices, i.e., conventional tillage (CT: two harrowings/rotavator, two plowings using a tine cultivator, and one field leveling using a wooden plank), whereas some farmers adopted zero tillage (ZT) for growing rice and wheat. In the CT system, rice was established by transplanting 25–30-day-old seedlings in puddled (wet tillage) soil. In the CT system, wheat was established either by broadcasting the seeds in the field after land preparation or by drilling using a seed-cum-fertilizer drill. In the ZT system, both rice and wheat were seeded using a zero-till planter or a turbo Happy Seeder^32^ without preparatory tillage. Depending upon water availability and farmers’ preference, some farmers kept their rice field continuously flooded, whereas some followed alternate wetting and drying cycles. In Punjab and Haryana, rice fields received 20–25 irrigations per season, whereas in Bihar, farmers applied only 4–8 irrigations depending upon rainfall. In general, wheat received four irrigations of 6–7 cm each at 20–25, 45–50, 75–80 and 110–120 days after sowing.

We calculated NE-based fertilizer recommendations using the farm management information provided by the farmers supplemented with soil and climatic condition of the field. NE-based fertilizer recommendations varied from farm to farm depending upon soil type, cropping history and management practices, whereas a farmer’s fertilizer practice was as per his/her prevailing practice. Farmers’ fertilizer management practices also varied from farm to farm depending upon farmers’ knowledge of fertilizer management, their purchasing power and so on. For both crops, the total amounts of P_2_O_5_ and K_2_O and 15–20% of the N were applied as basal fertilizer using di-ammonium phosphate and muriate of potash. The remaining amount of N was top-dressed in two equal splits 20–25 and 40–50 days after seeding/transplanting using urea under both NE and FFP. Figure 1 shows the average N, P_2_O_5_ and K_2_O rates under NE and FFP for both crops in both agro-ecologies and Supplementary Fig. S1 presents their pairwise distribution.

### Data collection

We recorded and compiled all the management practices in each farmer’s field, such as tillage and residue management, nutrient and water management, as well as crop protection. We obtained climate information (temperature and rainfall) about the farm from the nearest agricultural science center. We obtained site specific soil data such as texture, soil organic carbon, soil pH and bulk density from the International Soil Reference and Information Centre (https://www.isric.org/explore/soilgrids)^33^. We also recorded the amount of fuel and electricity consumed for various farm operations during the entire crop cycle. At maturity, we recorded the grain yield (at 13% moisture content) by harvesting three 3 m^2^ quadrates in each plot.

### Estimation of GHG emissions and global warming potential

We estimated GHG emissions from each plot using the CCAFS Mitigation Options Tool^34^, hereafter referred to as CCAFS-MOT, which combines several empirical models to estimate GHG emissions from different land uses. The tool recognizes context specific factors that influence GHG emissions such as soil and climate, production inputs and management practices. To estimate total GHG emissions from the production systems, i.e., global warming potential (GWP), we converted all GHG emissions into CO_2_ equivalents (CO_2_e) using the global warming potential (over 100 years) of 28 and 265 for CH_4_ and N_2_O, respectively^35^. We then divided total GWP by grain yield to determine GHG emission intensity.

### Estimation of fertilizer costs and income from crop yield

As everything except nutrient management was similar within the comparison pair, we only used the fertilizer cost and income from yield for comparison between NE and FFP. The year-wise fertilizer cost and price of grains that were used for the economic analysis is provided in Supplementary Table 2. The fertilizer cost was estimated by using the market rate of the respective fertilizer for the respective years obtained from the Fertilizer Association of India (https://www.faidelhi.org/; Supplementary Table 2). We calculated the income from grain yield by multiplying the total grain yield with minimum support price (MSP) for the respective years (Supplementary Table 2). The MSP is an agriculture product price set by the Food Corporation of India (FCI) to purchase directly from the farmers (http://fci.gov.in).

### Statistical analysis

We conducted a paired t-test comparison of the variables of interest using Costat Software^36^. As we had pair comparisons of NE versus FFP in each farmer’s field, the paired t-test is appropriate for examining the difference in means. Once the effect of NE over FFP in terms of fertilizer rate, yield, GHG emissions, fertilizer cost and income were determined through the paired t-test, those variables were also subjected to meta-analysis to determine the influence of various agro-climatic conditions and management factors (e.g., crop types, agro-climatic zone, farmers’ fertilizer rate) on the effectiveness of NE over FFP. For this, we considered each of the on-farm comparison trials characterized by crop, location, year, soil properties, management information and so on, as a data point. All the trials within a location that were similar in the above-mentioned characteristics constituted a study (a village had one or more studies), and all pair-comparison trials within the specific study were considered replications and used to calculate the standard deviation and effect size in meta-analysis. We performed meta-analysis using MetaWin 2.1 in two stages^37,38^. At first, we calculated the effect size for each study as the natural log of the response ratio (lnR) using the following equation^39^:

$$Effect \; size = lnR=\mathit{ln}\left[\frac{{X}_{NE}}{{X}_{FFP}}\right]$$

where X_NE_ is the mean of response variables (yield, N-rate, GHG emission intensity, global warming potential) due to NE, and X_FFP_ is the mean of these variables in FFP. This ratio is comparable between the studies, while the logarithmic transformation ensures that variability in the ratio's denominator has no greater influence on the metric than variability in its numerator.

We then combined the effect sizes from the studies using a mixed-effect model to calculate the cumulative effect size and the 95% confidence intervals (CIs) through bootstrapping with 4999 iterations^40^. The mixed-effect model is a random-effect meta-analytic model for categorical data^37^, assuming random variation among studies within a group and fixed variation between groups. We considered the cumulative effect significant if the CIs did not overlap with zero and the effect sizes among the categories significantly different if their CIs did not overlap. For ease of interpretation, we back-transformed the results and reported them as the percentage change caused by NE in relation to FFP. We considered the difference significant only when p values were < 0.05.

## Supplementary Information

Supplementary information.

## References

[1] Ross K (2019). Enhancing NDCs: Opportunities in Agriculture.

[2] Carlson KM (2017). Greenhouse gas emissions intensity of global croplands. Nat. Clim. Change..

[3] Kanter DR, Zhang X, Mauzerall DL, Malyshev S, Shevliakova E (2016). The importance of climate change and nitrogen use efficiency for future nitrous oxide emissions from agriculture. Environ. Res. Lett..

[4] IPCC (2019). IPCC special report on climate change, desertification, land degradation, sustainable land management, food security, and greenhouse gas fluxes in terrestrial ecosystems. Summary for policymakers.

[5] Reynolds TW (2015). Environmental impacts and constraints associated with the production of major food crops in Sub-Saharan Africa and South Asia. Food Secur..

[6] Spiertz JHJ (2009). Nitrogen, sustainable agriculture and food security: A review. Sustain. Agric..

[7] Garnett T (2013). Sustainable intensification in agriculture: Premises and policies. Science.

[8] Sutton, M. A. *et al. Our nutrient world: the challenge to produce more food and energy with less pollution*. (Global Overview of Nutrient Management. Centre for Ecology and Hydrology, Edinburgh on behalf of the Global Partnership on Nutrient Management and the International Nitrogen Initiative, 2013).

[9] Good AG, Beatty PH (2011). Fertilizing nature: A tragedy of excess in the commons. PLoS Biol..

[10] Buresh, R. J. & Witt, C. Site-specific nutrient management. In *Fertilizer Best Management Practices: Gneral principles, strategy for their adoption and voluntary initiatives vs regulations. Paper presented at IFA International Workshop on Fertilizer Best Management Practices, 7–9 March 2007, Brussels, Belgium* (ed. IFIA) (International Fertilizer Industry Association, 2007).

[11] Dobermann, A. & Witt, C. The evolution of site-specific nutrient management in irrigated rice systems of Asia. In *Increasing productivity of intensive rice systems through site-specific nutrient management* (eds. Dobermann, A., Witt, C. & Dawe, D.) 410 (Science Publisher Inc., and International Rice Research Institute (IRRI), 2004).

[12] Shapiro CA (2013). Using a Chlorophyll Meter to Improve N Management.

[13] LCC. Leaf Color Chart (LCC). (2020). http://www.knowledgebank.irri.org/step-by-step-production/growth/soil-fertility/leaf-color-chart.

[14] Bijay-Singh (2015). Site-specific fertilizer nitrogen management in irrigated transplanted rice (*Oryza sativa*) using an optical sensor. Precis. Agric..

[15] Sapkota TB, Nagothu US (2016). Precision nutrient management under conservation agriculture-based cereal systems in South Asia. Climate Change and Agricultural Development: Improving Resilience through Climate Smart Agriculture, Agroecology and Conservation.

[16] Pampolino MF, Witt C, Pasuquin JM, Johnston A, Fisher MJ (2012). Development approach and evaluation of the Nutrient Expert software for nutrient management in cereal crops. Comput. Electron. Agric..

[17] Xu X (2017). Methodology of fertilizer recommendation based on yield response and agronomic efficiency for rice in China. Food Crop. Res..

[18] Jat RD (2018). Conservation agriculture and precision nutrient management practices in maize-wheat system: Effects on crop and water productivity and economic profitability. Food Crop. Res..

[19] Sapkota TB (2014). Precision nutrient management in conservation agriculture based wheat production of Northwest India: Profitability, nutrient use efficiency and environmental footprint. Food Crop. Res..

[20] Heffer, P., Gruere, A. & Roberts, T. *Assessment of Fertilizer Use by Crop at the Global Level*. *International Fertilizer Association (IFA) and International Plant Nutrition Institute (IPNI)* **5**, (2017).

[21] Farnworth CR, Stirling C, Sapkota TB, Jat ML, Misiko M (2017). Gender and inorganic nitrogen: What are the implications of moving towards a more balanced use of nitrogen fertilizer in the tropics ?. Int. J. Agric. Sustain..

[22] Singh VK, Dwivedi BS, Shukla AK, Chauhan YS, Yadav RL (2005). Diversification of rice with pigeonpea in a rice–wheat cropping system on a TypicUstochrept: Effect on soil fertility, yield and nutrient use efficiency. Food Crop. Res..

[23] Xu X (2016). Narrowing yield gaps and increasing nutrient use efficiencies using the Nutrient Expert system for maize in Northeast China. Food Crop. Res..

[24] Sapkota TB (2020). Identifying optimum rates of fertilizer nitrogen application to maximize economic return and minimize nitrous oxide emission from rice–wheat systems in the Indo-Gangetic Plains of India. Arch. Agron. Soil Sci..

[25] Reay DS (2012). Global agriculture and nitrous oxide emissions. Nat. Clim. Change.

[26] Albanito F (2017). Direct nitrous oxide emissions from tropical and sub-tropical agricultural systems—A review and modelling of emission factors. Sci. Rep..

[27] Zhang JJ (2018). Nutrient expert improves nitrogen efficiency and environmental benefits for winter wheat in China. Agron. J..

[28] Cui Z (2008). On-farm evaluation of an in-season nitrogen management strategy based on soil Nmin test. Food Crop. Res..

[29] Sapkota TB (2019). Cost-effective opportunities for climate change mitigation in Indian agriculture. Sci. Total Environ..

[30] Bordoloi N, Baruah KK, Hazarika B (2020). Fertilizer management through coated urea to mitigate greenhouse gas (N_2_O) emission and improve soil quality in agroclimatic zone of Northeast India. Environ. Sci. Pollut. Res..

[31] Tewatia RK, Chanda TK, Abrol YP (2017). Trends in fertilizer nitrogen proudction and consumption in India. The Indian Nitrogen Assessment: Sources of Reactive Nitrogen, Environmental and Climate Effects, Management Options and Policies.

[32] Sidhu HS (2015). Development and evaluation of the Turbo Happy Seeder for sowing wheat into heavy rice residues in NW India. Food Crop. Res..

[33] Hengl T (2017). SoilGrids250m: Global gridded soil information based on machine learning. PLoS ONE.

[34] Feliciano D, Nayak DR, Vetter SH, Hillier J (2017). CCAFS-MOT—A tool for farmers, extension services and policy-advisors to identify mitigation options for agriculture. Agric. Syst..

[35] IPCC. *Climate Change 2013. The Physical Science Basis. Working Group I contribuiton to the Fifth Assessment Report of the Intergovernmental Panel on Climate Change. Chapter 8: Anthropogenic and Natural RAdiative Forcing. Intergovernmental Panel on Climate Chang*. (2013).

[36] CoHort, S. CoHort Software. *Monterey, CA. USA* (2017). http://www.cohort.com. Accessed 17 July 2017.

[37] Rosenberg MS, Adams DC, Gurevitch J (2000). MetaWin: Statistical Software for Meta-analysis. Version 2.0.

[38] Chakraborty D (2017). A global analysis of alternative tillage and crop establishment practices for economically and environmentally efficient rice production. Sci. Rep..

[39] Hedges LV, Gurevitch J, Curtis PS (1999). The meta-analysis of response ratios in experimental ecology. Ecology.

[40] Adams DC, Gurevitch J, Rosenberg S (1997). Resampling tests for meta-analysis of ecological data. Ecology.

