# Supplementary material for: Crop nutrient management using Nutrient Expert improves yield, increases farmers’ income and reduces greenhouse gas emissions
Source: Sci Rep. 2021 Jan 15;11:1564. doi: 10.1038/s41598-020-79883-x (PMC7810863; doi:10.1038/s41598-020-79883-x)
Supplement: Supplementary file 1 — Supplementary information. [file 41598_2020_79883_MOESM1_ESM.docx]

Supplementary materials for

**Crop nutrient management using Nutrient Expert improves yield, increases farmers’ income and reduces greenhouse gas emissions**

Tek B Sapkota^1^, Mangi L Jat^2*^, Dharamvir S Rana^3^, Arun Khatri-Chhetri^4,^ Hanuman S Jat ^5^, Deepak Bijarniya^6^, Jhabar M Sutaliya ^7^, Manish Kumar^8^, Love K Singh^8^ Raj K Jat^9^, Kailash Kalvaniya^2^, Gokul Prasad^2^, Harminder S Sidhu^8^, Munmun Rai^2^, T Satyanarayana^10^, Kaushik Majumdar^11^

^1^International Maize and Wheat Improvement Centre (CIMMYT, El Batan, Mexico

^2^International Maize and Wheat Improvement Centre (CIMMYT, New Delhi, India.

^3^International Rice Research Institute (IRRI), NASC complex, New Delhi 110012, India

^4^CGIAR Research Program on Climate Change, Agriculture and Food Security (CCAFS), CIAT-Bioversity alliance, Cali, Colombia.

^5^ICAR-Central Soil Salinity Research Institute (CSSRI), Karnal, Haryana, India

^6^International Maize and Wheat Improvement Centre (CIMMYT), CSSRI, Karnal, India

^7^CCS Haryana Agriculture University, Hisar, Haryana, India

^8^International Maize and Wheat Improvement Centre (CIMMYT)-, Borlaug Institute for South Asia (BISA), Ludhiana, Punjab , India

^9^International Maize and Wheat Improvement Centre (CIMMYT)-Borlaug Institute for South Asia (BISA), Pusa, Samastipur Bihar, India

^10^International Plant Nutrition Institute (IPNI), Gurgaon 122001, Haryana, India

^11^African Plant Nutrition Institute (IPNI), Benguérir, Morocco

*Corresponding/presenting author email address: [M.Jat@cgiar.org](mailto:t.sapkota@cgiar.org); Phone # +91 9999108787


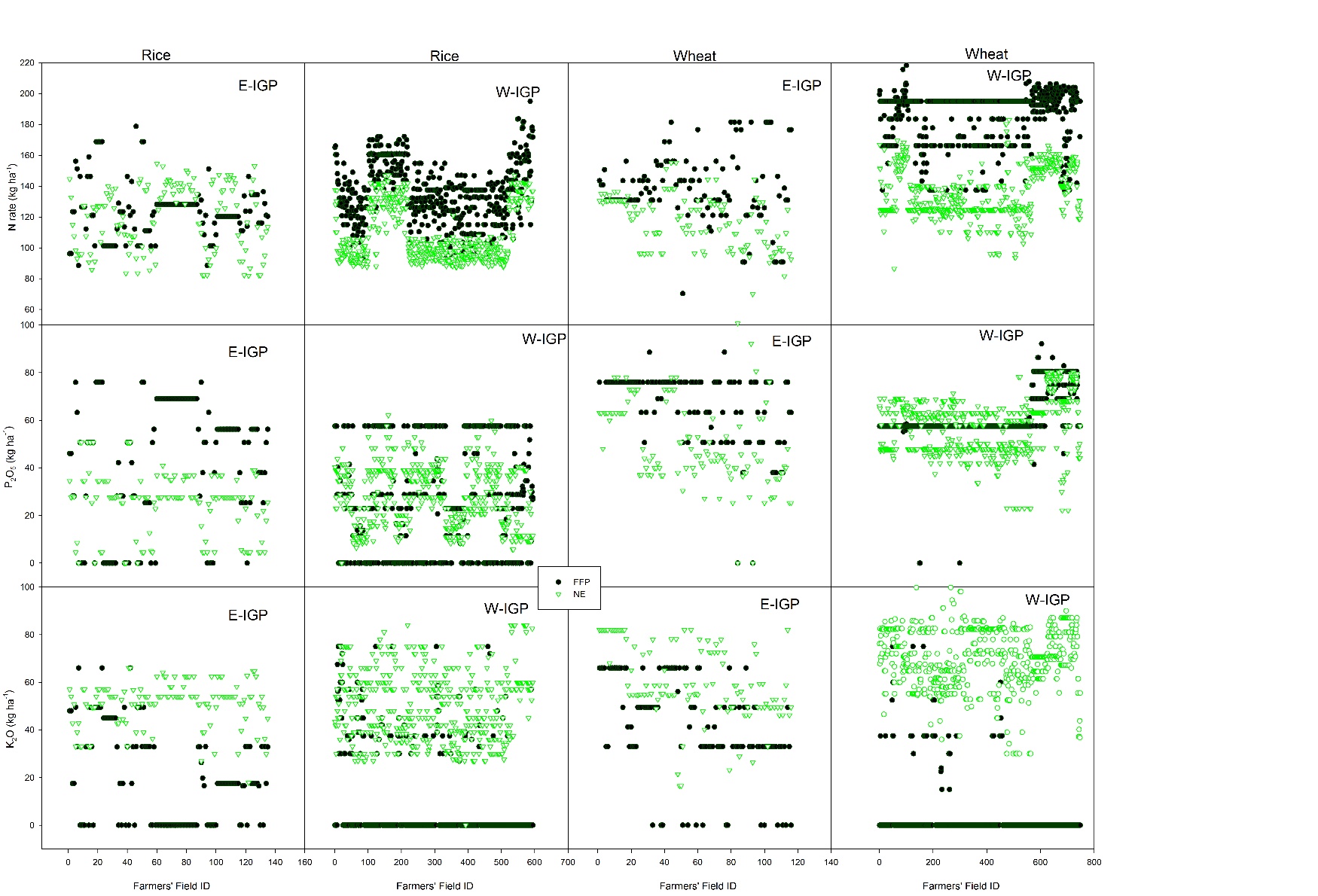


Fig. S1. Rate of nitrogen (N), phosphorus (P_2_O_5_) and potash (K_2_O) application for rice and wheat under Nutrient Expert (NE) and Farmers’ Fertilizer Practice (FFP) in the study areas. EIGP = Eastern Indo-Gangetic Plains. W-IGP = Western Indo-Gangetic Plains.


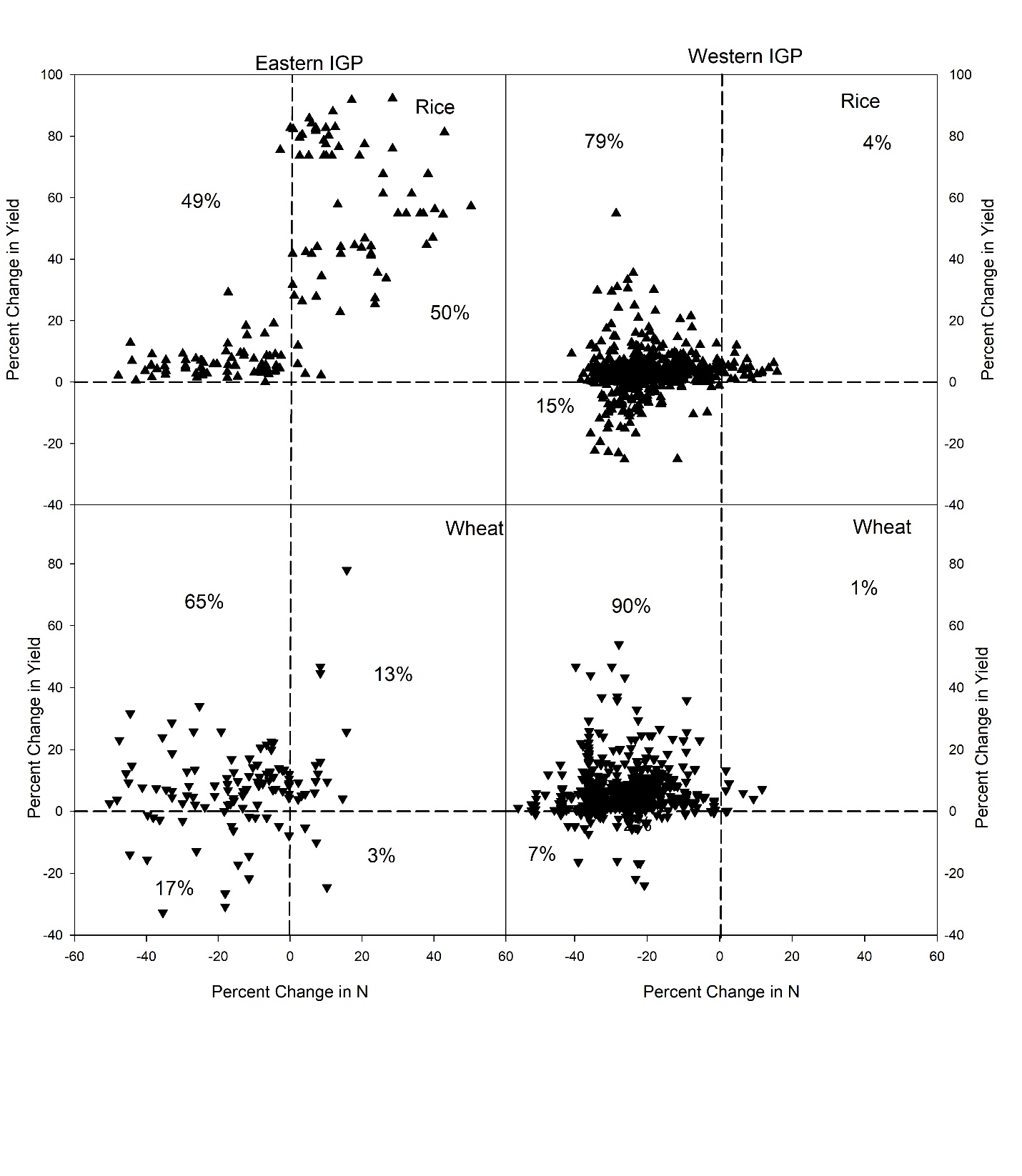


Fig. S2. Change in N input and corresponding change in crop yield due to NE-based fertilizer management over farmers’ fertilizer practice in rice and wheat in the study area. IGP = Indo-Gangetic Plains.


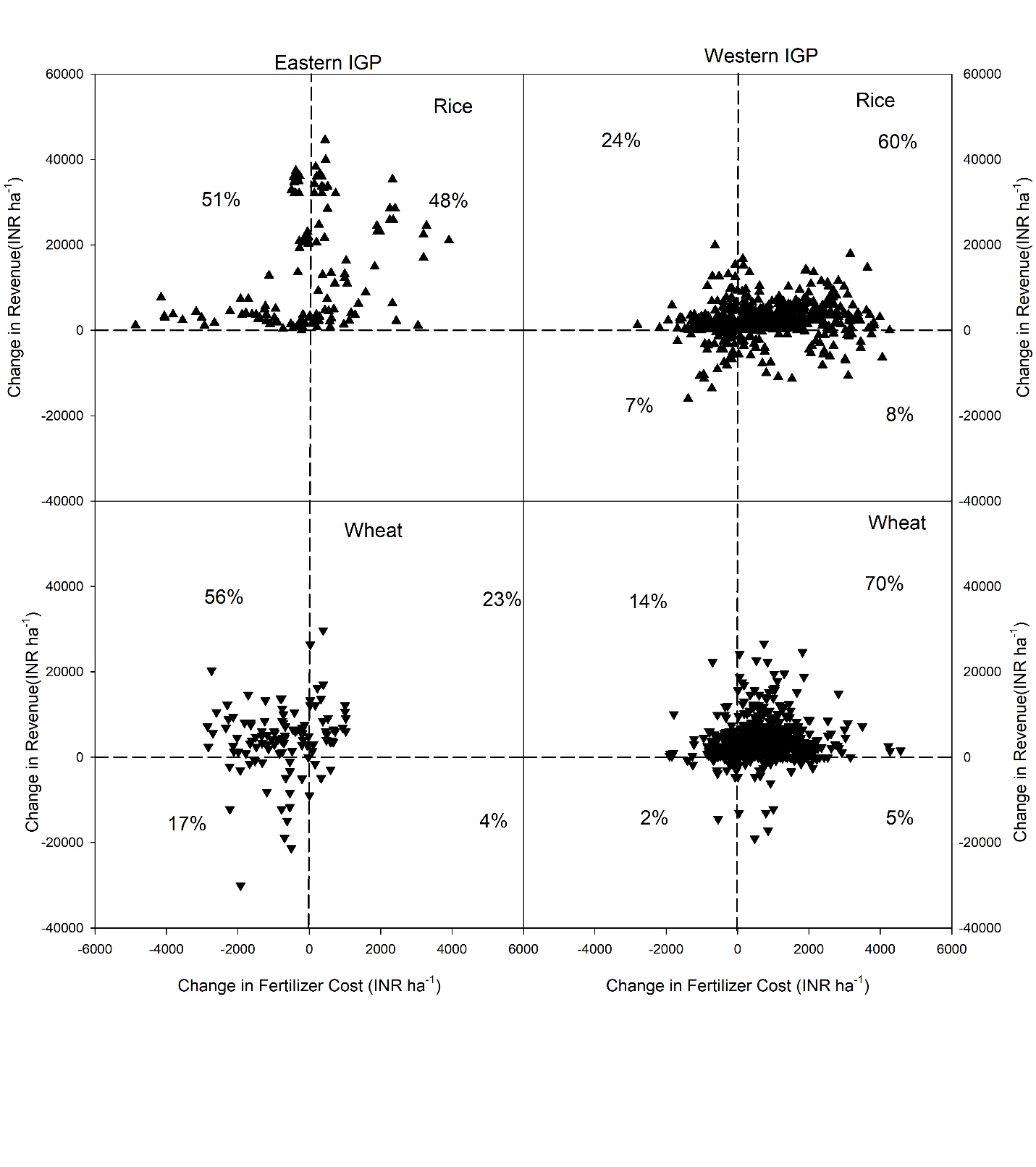
 Supplementary Fig. S3. Change in fertilizer cost and corresponding change in crop yield revenue due to NE-based fertilizer management compared with farmers fertilizer practice in rice and wheat in the study area. IGP = Indo-Gangetic Plains.


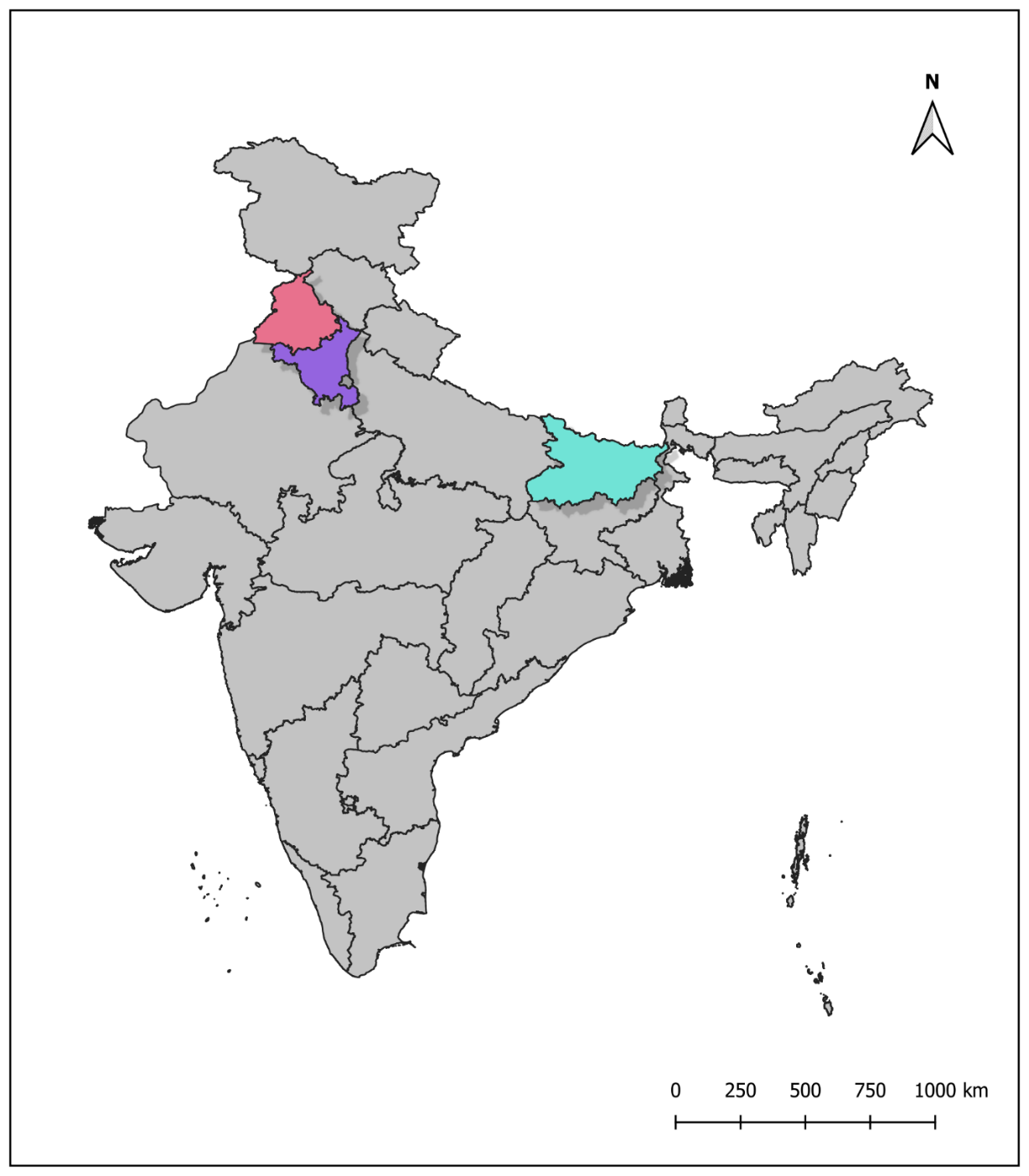


Figure S4. The study locations within Indo-Gangetic Plains of India. Two states i.e. Haryana and Punjab lie in Western IGP and Bihar lies in Eastern IGP. The map was generated using QGIS (version 3.10; <https://www.qgis.org/en/site/>). Please see supplementary Table 1 for basic agro-ecological characteristics of the study sites.

Supplementary Table 1: Basic agro-ecological characteristics of the study sites

| Particulars | Punjab | Haryana | Bihar |  |
| --- | --- | --- | --- | --- |
| Latitude | 30.52268°-30.9944° N | 29.27339°-29.9816° N | 25.44578° -25.9237°N |  |
| Longitude | 75.69041°-76.51383° E | 70.91994°-77/97899° E | 85.1908°-85.7271° E |  |
| Crops included in the study | Rice & Wheat | Rice & Wheat | Rice &Wheat |  |
| Average annual Rainfall (mm) | 544-870 | 650-970 | 1350 |  |
| Agro-ecology | Warm arid and semiarid subtropics | Warm arid and semiarid subtropics | Warm sub humid subtropics |  |
| Soil texture | Loam-Silt loam | Loam-Silt loam | Silt Loam to Clay loam | |
| Soil pH | 7.8-7.9 | 7.4 - 8.3 | 7.6-8.1 |  |
| Soil organic carbon (%) | 0.4-0.8 | 0.24 - 0.72 | 0.49-0.87 |  |
| Soil bulk density (g/cm3) | 1.45-1.55 | 1.35-1.65 | 1.5-1.58 |  |
| Rice area (‘000’ ha) | 2825 | 1243 | 2845 |  |
| Wheat area (‘000’ ha) | 3510 | 2504 | 2100 |  |

Supplementary Table 2: Year-wise cost of key inputs and outputs used for economic analysis during rice and wheat growing season^†^

| Particulars | 2013-14 | 2014-15 | 2015-16 | 2016-17 |
| --- | --- | --- | --- | --- |
| Price of wheat grain (INR Mg^-1^) | 14000 | 14500 | 15250 | 16250 |
| Price of rice grain (INR Mg^-1^) | 13100 | 13600 | 14100 | 14700 |
| Cost of N (INR kg^-1^) | 11.65 | 11.65 | 11.65 | 12.17 |
| Cost of P_2_O_5_ (INR kg^-1^) | 46.96 | 48.70 | 43.27 | 45.60 |
| Cost of K_2_O (INR kg^-1^) | 27.5 | 26.67 | 18.33 | 26.67 |

^†^Source:

Fertilizer Association of India (<https://www.faidelhi.org/>)

Food Corporation of India (<http://fci.gov.in>)
